# The Effect of a Turkey Berry (*Solanum torvum*)-Fortified Biscuit on the Hemoglobin Level and Cognitive Performance of Adolescent Females in the Ahafo Region of Ghana: A Pilot Study

**DOI:** 10.1155/2023/1388782

**Published:** 2023-10-20

**Authors:** Abigail Owusuaa Appiah, Marina Aferiba Tandoh, Pepertual Suglo Puotege, Anthony Kwaku Edusei

**Affiliations:** ^1^Department of Biochemistry and Biotechnology (Human Nutrition and Dietetics), College of Science, Kwame Nkrumah University of Science and Technology, PMB, Kumasi, Ghana; ^2^Department of Health Promotion and Disability Studies, School of Public Health, Kwame Nkrumah University of Science and Technology, Kumasi, Ghana

## Abstract

Anemia is a public health problem that affects about 50% of adolescent females in sub-Saharan Africa. Anemia can negatively affect the overall growth, cognitive performance, and productivity of school-going adolescents. This pilot study assessed the impact of *Solanum torvum-*fortified biscuits on hemoglobin levels and cognitive performance of school-going adolescent females. A cluster randomized controlled, open labeled trial was undertaken among four basic schools in the Ahafo Region of Ghana. Out of the 169 participants, 151 (intervention = 83, control = 68) adolescent females completed the trial. The intervention and control groups were made up of two schools each. The participants in the intervention and control groups received a total of 30 turkey berry-fortified biscuit supplementation or 30 placebos for a period of six weeks, respectively. The hemoglobin levels and cognitive test scores were obtained before and after the intervention. The number of anemic cases and low cognitive performance among the female adolescents in the intervention group reduced by 23.8% and 8.7%, respectively. There was a positive impact on the hemoglobin levels and cognitive performance of the intervention group. For every unit of turkey berry-fortified biscuit consumed by the intervention group, there was a 0.945 unit increase in hemoglobin level (*p* = 0.001) and a 2.796 unit increase in cognitive performance (*p* = 0.005). The turkey berry-fortified biscuit significantly reduced anemia prevalence and improved cognitive performance among the adolescent girls. Therefore, its potential in the management of anemia and improvement of cognition could be explored.

## 1. Introduction

Anemia is a condition characterized by low levels of an iron-rich protein known as hemoglobin [1, 2]. Hemoglobin gives blood its red colouration and carries oxygen from the lungs to other parts of the body for metabolic activities [1, 2]. Anemia is a public health problem, with 89% of the world's anemia burden occurring in developing countries, of which Ghana is part [3]. The prevalence of anemia in adolescents worldwide is 15%, with 27% from developing countries [4]. The most common cause of anemia is iron deficiency, which accounts for about 50% cases of anemia [3]. Globally, it is estimated that the major cause of morbidity and mortality in adolescent females is iron deficiency anemia [5]. Anemia negatively affects the cognitive development and academic performance of adolescents [3]. Anemia can also result in low work productivity, pregnancy complications, and poor birth outcomes such as low birth weight, premature birth, and stillbirth which have a negative impact on the economy [3].

According to the World Health Organization (WHO), adolescence is the period between 10 and 19 years of age [6]. During this period, the requirement of energy and nutrients such as protein, iron, and calcium is high for desired growth [2]. Malnutrition in adolescent females is directly linked to chronic diseases and anemia [1].

The increase in body mass and blood volume and the onset of menstruation which causes about 12.5–15 mg loss of iron monthly increase the iron requirement for adolescent females to 8 mg–15 mg, and that puts them at risk of iron deficiency anemia [3, 7]. Concurrently, adolescent girls in Ghana have poor dietary intakes that affect their nutritional intake of hemoglobin-forming nutrients such as iron, folate, vitamin B_12_, and protein [8]. Inadequate dietary iron intake, worm infestation, infections, disorders of hemoglobin synthesis, and teenage pregnancy further put adolescents at risk of anemia [9]. Low dietary diversity scores in adolescents also predispose them to iron and other micronutrient deficiencies [10].

An intervention in the form of school-based Girl Iron Folic Acid Supplementation (GIFTS) (60 mg elemental iron and 400 *μ*g of folic acid) was initiated by the Ministry of Health together with the Ghana Education Service and other agencies in five regions of Ghana [11]. The main aim of the Girl Iron Folic Acid Supplementation was to reduce and prevent anemia in adolescent females aged 15–19 [12]. However, a study in 2019 revealed that only 26% of the adolescent females took a fraction of the tablets [12]. Most adolescents together with their parents perceived the tablet to be a contraceptive and refused to partake in the GIFTS program [12]. This is a clear indication that a community-based, culturally acceptable, and cost-effective solution to micronutrient deficiencies is necessary.

Turkey berries (*Solanum torvum*), locally known in Ghana as “kwahunsusua,” are locally grown plants used for diverse health benefits in Ghana. Cooked fruits of *Solanum torvum* are traditionally used as an adjunct therapy for people with anemia, and the ripened fruits are also used in preparing hemopoietic agents [13]. Turkey berry is used in Ghana for the preparation of stews and soups. The presence of iron in the fruit is an indication that the fruit has hematinic properties [14].

A proximate analysis of turkey berries showed that it contained carbohydrate (7.04%), protein (2.32%), fat (0.28%), crude fibre (3.99%), ash (0.14%), moisture (86.23%), calcium (221.58 mg/kg), iron (76.87 mg/kg), vitamin C (2.69 mg/kg), zinc (21.46 mg/kg), manganese (19.47 mg/kg), copper (2.65 mg/kg), and vitamin A (0.08 mg/kg) [15]. The use of *Solanum torvum* (turkey berries) has not been extensively explored even though it contains important nutrients.

This study is a follow-up of our previous work [16]. A nutrition intervention was developed for the same participants at the same study site to answer a different research question which was to measure the impact of our nutrition intervention on the baseline data obtained from the participants. The main objective of this pilot study was to determine the efficacy of turkey berry-fortified biscuit in improving the hemoglobin level and cognitive performance of school-going adolescent females.

## 2. Materials and Methods

### 2.1. Materials, Reagents, and Equipment

The reagents used in this study included the following:

- Nitric acid (HNO_3_) 69% (*w*/*v*), AnalaR NORMAPUR analytical reagent, manufactured by VWR Chemicals, Radnor (Pennsylvania)
- Sulfuric acid (H_2_SO_4_) 95–98% (*w*/*v*), ExpertQ, ACS, ISO, max. 0.0000005% Hg, manufactured by Scharlab S.L., Gato Perez (Spain)

The following equipment was also employed in this study:

- MS105DU Electronic analytical balance, 120/42 g capacity, 0.01/0.1 mg readability, manufactured by Mettler Toledo (Switzerland) used for weighing samples
- 50 ml graduated digestion tube, manufactured by Thermo Electron LED GmbH, Langenselbold, (Germany), made of thick-walled borosilicate glass with heavy-duty retention lips and contoured bottoms for optimal heat transfer and minimal thermal shock used for sample digestion

All the reagents and equipment listed above were obtained from the Food Science Laboratory of the Kwame Nkrumah University of Science and Technology.

### 2.2. Study Setting and Population

This pilot study was conducted in two towns (Tanoso and Yamfo) in the Tano North Municipal District of the Ahafo Region of Ghana. There are about 10 government-owned schools in these communities. The study participants were recruited from two government-owned schools in each of the towns (Yamfo and Tanoso). Adolescent females on a regular iron-folic acid tablet or any hematinic for three months preceding this study were excluded from the study. This study was conducted in the Ahafo Region, one of the five regions for the GIFTS program, to promote an alternative method in the management and prevention of the high prevalence of anemia among adolescent girls.

### 2.3. Study Design and Sample Size

**Figure 1:**
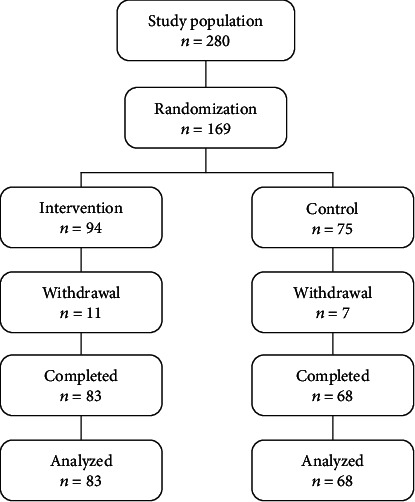
Flow diagram for the turkey berry-fortified biscuit trial.

A sample size of 260 was determined using Cochran's formula [17]. A cluster randomized control trial was conducted among adolescent females between the ages of 10–19 years in four selected government schools. The parents of 169 adolescent females consented for their children to take part in the study. A total of 151 out of the 169 adolescent females recruited were able to complete the study. The four schools were randomized into control and intervention arms. Randomization of the schools was done through balloting. Each of the intervention and control groups was made up of two schools from each town. The intervention and control groups were made up of 55.6% and 44.4% participants, respectively.  Figure 1 is the flow diagram of the study.

### 2.4. Data Collection

Information was obtained from the participants using a structured questionnaire. Data collected at baseline included biochemical data (hemoglobin level, microhematuria, and proteinuria) and cognitive test scores. A follow-up data was collected 6 weeks after the intervention.

### 2.5. Dietary Assessment

The nine (9) aggregated food groups by the Food and Agriculture Organization (FAO) of the United Nations and United States Agency for International Development's (USAID) food and nutrition technical assistance III project (FANTA) were adopted for the dietary assessment of the participants. The questionnaire was modified to reflect locally consumed foods in Ghana. The nine food groups include starchy staples; dark green leafy vegetables; other vitamin A-rich fruits and vegetables; other fruits and vegetables; organ meat; meat and fish; egg; legumes, nuts, and seeds; and milk and milk products [18].

A 24-hour recall was taken to help in the nutrition assessment. The participants were asked to recall and describe all foods and drinks/beverages consumed in the previous 24 hours. Quantities of foods were estimated with handy measures. A food group was recognized if at least 15 g of the food group was consumed. The participants were told to maintain their normal dietary intake throughout the study.

### 2.6. Preparation of the Turkey Berry Flour

The turkey berry fruits were purchased from Ejisu and Adum, local markets located at Kumasi, a suburb of the Ashanti Region in Ghana. Purchased turkey berry fruits were sent to the Department of Horticulture, Kwame Nkrumah University of Science and Technology (KNUST), in Kumasi, where the berries were separated from the stalks and washed. Washed berries were air dried for one hour before pulverized into smaller units using an electric crusher and laid out in baking trays for oven drying. The oven drier was set to 60°C and preheated for 10 minutes before pulverized berries were placed into it for further drying. Sixty degrees Celsius (60°C) is efficient in drying the berries with minimal losses in appearance and texture. The berries were stirred periodically to ensure that there was even drying. The total amount of time taken for total drying of berries was 36 hours. Dried berries were taken out of the oven drier and scooped using a spatula into an electric grinder for further processing. The ground berries were weighed using an electronic balance and packaged into ziplock bags for further use. 5 g of the powdered turkey berry was added to each biscuit and baked together with other main ingredients such as flour, eggs, vegetable oil, and sugar.

### 2.7. Preparation of Biscuits

The ingredients used for the preparation of a hundred (100) pieces of the biscuits included turkey berry flour (500 g/two cups), soft flour (1,150 g), sugar (345 g), margarine (2,724 g), milk powder (Nido) (104 g), egg white (2), vanilla essence (5 ml), nutmeg (10 g), and water (500 ml).

The oven was preheated to about 230°C.

All the dry ingredients such as soft flour, turkey berry flour, sugar, nutmeg, and milk powder were mixed together (for the control group, turkey berry flour was not added).

The wet ingredients such as margarine, egg, vanilla essence, and water were then added and mixed together with the dry ingredients to form a dough. The dough was rolled out and cut into about half-inch thickness round biscuits.

The biscuits were then coated with egg white and baked for 30-45 minutes. Fresh biscuits were baked, packaged, and delivered to the participants (after the baseline data was taken) every school day for a period of six weeks.

The participants were required to consume one biscuit, five times in a week for six (6) weeks. It was ensured that the participants consumed the biscuits right after they received them to prevent sharing them with friends or discarding them. The intervention group received biscuits fortified with turkey berry while the control group received the same number of biscuits which had not been fortified. Each biscuit for the intervention group contained 5 g of turkey berry flour.

### 2.8. Mineral Analysis

Iron is a major mineral in the production of hemoglobin, but nutrients do not work in isolation; minerals such as zinc and magnesium play important roles in blood formation. Zinc serves as a catalyst for many enzymes that are needed for red blood cell production. Magnesium is also important in the hematopoietic system. In this study, we analyzed the amount of minerals such as iron (Fe), zinc (Zn), magnesium (Mg), potassium (K), and calcium (Ca) [15].

The digestion of the plain and turkey berry-fortified biscuit samples was done by weighing 1 gram of each sample into a digestion tube, and 1 ml of distilled water was added and 8 ml of HNO_3_. Then, 5 ml of H_2_SO_4_ was added and subjected to heat for 30 minutes at 200°C. The digested samples were diluted with 50 ml deionized water, and atomic absorption spectrometer (AAS) was used to determine minerals like iron (Fe), zinc (Zn), calcium (Ca), and magnesium (Mg), while potassium (K) was determined using a flame photometer [19].

Calculation:

(1) $$\begin{array}{c} \text{Conc}.\left(\text{Fe},\text{Zn},\text{Ca},\text{Mg}\right)\left(\text{mg}/\text{kg}\right)=\frac{\text{conc}.\text{recorded from AAS}\times \text{nominal volume}}{\text{sample weight }\left(\text{g}\right)}, \end{array}$$

where nominal volume is 50 ml and sample weight is 1.00 g.

### 2.9. Hemoglobin

The hemoglobin (Hb) levels of all participants were determined using a portable hemoglobin meter URIT 12 manufactured by Medical Electronic Group Company Limited in China. The test strip utilizes dry chemistry colorimeter method. The capillary blood samples of the participants were obtained by pricking their middle finger and placing a drop of their blood onto the hemoglobin meter strip. The tip of the finger was wiped with methylated spirit before the prick. The Hb value of the participant was displayed on the screen of the hemoglobin meter and was recorded. When a drop of blood is applied to the test strip, blood immediately disperses within the membrane, contacting the reagent. The meter's optical detectors automatically measure the change in membrane reflectance. The intensity of the reflection is inversely proportional to the hemoglobin concentration.

Based on the WHO classification, Hb levels below 8 g/dl were classified as severe anemia, 8–10.99 g/dl as moderate anemia, 11–11.9 g/dl as mildly anemic, and Hb of 12 g/dl and above was classified as normal [20]. The hemoglobin levels were checked once, since a study by Alqershi et al. showed that there was no significant difference in parallel and duplicated hemoglobin readings using URIT-12 hemoglobin meter [21]. The baseline sample was taken on 4^th^ November 2019, and the end point sample was taken on 18^th^ December 2019.

### 2.10. Urinary Blood and Protein Loss

The urinary blood loss and protein loss were determined with a 10-parameter test strip. Urine samples were collected in dry and clean urine containers that allowed a complete immersion of the test strips from all participants except the participants who were menstruating at the time of sample collection. The reagent areas of the strips were completely immersed in the urine for approximately 3 seconds [22]. The reagent areas were then compared to the corresponding colour blocks on the colour chart [22]. The strips were matched carefully within 30 seconds to 2 minutes after contact with urine. Based on the colour change, the results were recorded [22].

### 2.11. Cognitive Assessment

Raven's Coloured Progressive Matrices was employed in the cognitive assessment of the participants at baseline and postintervention. The coloured progressive matrices contain three (3) sets of items, A, AB, and B, with twelve (12) items each, which sums up to thirty-six (36) items or questions. In each of the three sets, the questions are arranged in the order of ascending level of difficulty. The first item in the set A was explained and solved together with the participants. The participants were allowed to answer the questions at their own pace, and all participants finished within 30–45 minutes. The test booklet contained three sets of twelve problems (36 coloured questions), which measures fluid intelligence by problem-solving and abstract reasoning by analogy, and has been used extensively as a culturally fair test of intelligence [23] and has been used in Ghana in a study by Tandoh et al. [24] and by Annan et al. [25].

### 2.12. Data Analysis

Statistical Package for Social Sciences (SPSS) version 26 was used for data analyses. Continuous variables with normal distribution were expressed as mean ± SD (standard deviation) and compared between intervention groups using an independent sample *t*-test. Pearson's chi-square test was used to determine the differences in distribution for qualitative and categorical variables such as anemia status for the intervention and control groups. Simple linear regression analysis was performed to assess the impact of the turkey berry-fortified biscuit intervention (independent variable) on hemoglobin levels (dependent variable) and cognitive performance (dependent variable) of the participants. Statistical significance was considered at *p* < 0.05 at 95% confidence level.

## 3. Results

### 3.1. Mean Age, Prevalence of Anemia, and Cognitive Performance of Adolescent Females

Table 1 presents the mean age, prevalence of anemia, and cognitive performance of adolescent females. There was a significant difference in the mean ages of the intervention and control groups (*p* < 0.001). The mean hemoglobin level and cognitive performance score of the participants increased after the 6-week intervention. There were significant differences in the means of hemoglobin and cognitive performance of the intervention and control groups. The number of anemic cases reduced by 23.8% for the intervention group and increased by 25% for the control group 6 weeks postintervention. Most of the adolescent females passed the cognitive test at baseline and at postintervention.

### 3.2. Clinical and Biochemical Characteristics of the Participants

The clinical and biochemical characteristics of the adolescent females are summarized in Table 2. At baseline, 49.7% of the participants reported to have been diagnosed and treated for malaria one month prior to the study but at postintervention, 15.2% of the participants reported to have had malaria within the period of the study. The urinary blood loss test (hematuria) came out as negative and normal for most participants at baseline (94%) and postintervention (96.6%). At baseline, there were no significant differences observed in the distribution of urinary blood loss (*p* = 0.062) and protein loss (*p* = 0.141), between the control and intervention groups. At postintervention, there was a significant difference in the distribution of urinary protein loss (*p* = 0.003) between the control and intervention groups.

### 3.3. Consumption of the Nine Aggregated Food Groups by the Participants

Table 3 presents the consumption of the nine aggregated food groups by the participants. All the nine food groups were consumed by at least one participant except for organ meat which was consumed by none. All participants consumed starchy staples, while dark green leafy vegetable was consumed by 28.5% of the participants. Meat and fish were also consumed by 70.9% of the participants.

### 3.4. The Classification of Anemia of Adolescent Females at Baseline and Postintervention

**Figure 2:**
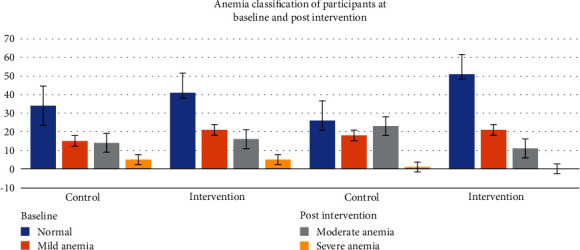
Anemia classifications of participants at baseline and postintervention.

The anemia classification of the adolescent females at baseline and postintervention is displayed in Figure 2. Seventy-six (50.3%) of the 151 participants were anemic at baseline. Of the 76 anemic participants, 55.3% were from the intervention group. The same numbers of participants from the intervention and control groups were severely anemic at baseline. Higher numbers of participants in the intervention group were moderately (16) and mildly (21) anemic compared to the control group (moderately (14) and mildly (15) anemic, respectively). At postintervention, the number of anemic participants in the intervention group decreased by 20.8% with none of them being severely anemic. The number of participants who were moderately anemic in the intervention group also decreased from 16 to 11. However, there was no change in the numbers who were mildly anemic. Conversely, the number of participants who were anemic in the control group increased from 34 to 42 even though the numbers who were severely anemic decreased. The number of participants moderately and mildly anemic from the control group also increased by 26.5% and 8.8%, respectively.

### 3.5. Impact of Turkey Berry-Fortified Biscuits on Hemoglobin Level and Cognitive Performance

Table 4 presents the impact of the turkey berry-fortified biscuits on hemoglobin levels of adolescent females. A simple linear regression was conducted to determine the impact on hemoglobin levels and cognitive performance of the intervention. For every unit of turkey berry-fortified biscuit consumed by the intervention group, there was a 0.945 unit increase in hemoglobin level and a 2.796 unit increase in cognitive performance. The increment in hemoglobin level and cognitive performance was significant at *p* values of 0.001 and 0.005, respectively.

### 3.6. The Mineral Composition of the Plain and Turkey Berry-Fortified Biscuit

Table 5 presents the mineral composition of the plain (100% wheat flour) and turkey berry-fortified biscuits. There were significantly higher levels of potassium (K), calcium (Ca), magnesium (Mg), zinc (Zn), and iron (Fe) in the turkey berry-fortified biscuit compared to the placebo. The most abundant and the least abundant mineral in both the placebo and the turkey berry-fortified biscuit was potassium and iron, respectively.

## 4. Discussion

Out of the 300 parental consent forms distributed, 169 (56%) parents consented for their children to participate in the study. Eighteen (18) participants withdrew from the study due to absenteeism. The overall mean age of the participants was 12.3 ± 1.76 years, which ranged from 10 to 17 years.

The number of participants with proteinuria and microhematuria for most of the classification at baseline was significantly reduced at the postintervention. All participants who had proteinuria and microhematuria at baseline did not have it at postintervention. Proteinuria and hematuria for most people resolve with repeated testing as shown in this study, and it could be attributed to the absence of renal disease and other underlining factors such as helminth infections [26, 27] and trauma which could be the risk factors for anemia [27].

The intervention had a positive impact on the hemoglobin levels of the participants. There was an 8.2% increment in the mean hemoglobin of the adolescent females in the intervention group. For every unit of turkey berry-fortified biscuit consumed by the intervention group, there was a 0.945 unit increase in hemoglobin level. The positive impact of the turkey berry-fortified biscuit on the hemoglobin levels of adolescent females is similar to a study conducted in India where supplementation of iron and folic acid-fortified biscuits improved the hemoglobin and serum iron levels of adolescent females significantly [28].

The prevalence of anemia among adolescent females in this study was reduced by 23.8% postintervention, and this is similar to an interventional study conducted among schoolchildren in Brazil where there was a reduction in the prevalence of anemia from 12.2% to 1.4% and from 11.5% to 4.2% after the schoolchildren were fed with iron-folic acid-fortified biscuit and iron-zinc-fortified biscuit, respectively, for 24 days within two months in Brazil [29].

A study conducted among school-age children (6–12years) in Nigeria also showed a significant increase in hemoglobin levels (15.5%), serum ferritin (28.3%), and serum vitamin A (26.3%) after pupils were fed with cowpea-fortified biscuits for 20 days [30]. The 24-hour recall of the participants in our study showed that the participants from both the intervention and control groups consumed common foods, and therefore, the significant increase in the hemoglobin level of the intervention group could be attributed to the consumption of turkey berry-fortified biscuits. The concentration of iron in the turkey berry fruits (76.87 mg/kg) justifies its hematinic effect and the reduction of anemia cases among the intervention group. Turkey berries are rich in nutrients required for growth and development, hence its positive impact on the participants' HB and cognitive performance.

There was a significant difference in the means of cognitive performance of the intervention group at baseline (24.19 ± 6.63) and postintervention (26.19 ± 6.19) of the study, and it could be attributed to the significant increment in the hemoglobin levels of the participants [31]. For every unit of turkey berry-fortified biscuit consumed by the intervention group, there was a 2.796 unit increase in cognitive performance. Lower hemoglobin level is associated with hypoxia [32]. Chronic hypoxia decreases cognitive performance and increases the risk of brain diseases [32].

A study conducted among adults in the United States showed that lower hemoglobin level was associated with slower working memory and smaller intracranial volume [31]. An increase in hemoglobin level increases cerebral perfusion, brain function, and cognitive performance. However, the improvement in cognitive functioning of adolescents may be due to differences in age and individual differences. These differences explain the variations in adolescents' knowledge, psychosocial maturity, reasoning (deductive, inductive, and decision-making), memory skills and processing constraints, and metacognitive orientation (reflection and evaluation of knowledge).

## 5. Conclusion

At postintervention, the number of anemic participants in the intervention group decreased by 20.8% with none of them being severely anemic. The hemoglobin level and cognitive performance of adolescent females in school were significantly increased by the consumption of turkey berry-fortified biscuits. The significant increment in hemoglobin levels of the participants and its positive impact on the cognitive performance of the participants in the intervention group is an indication that the biscuit is a good vehicle for fortification and turkey berry has the potential to improve hemoglobin level. Turkey berry-fortified biscuits could therefore be included in the schools' menus as snacks.

## Figures and Tables

**Table 1:** Mean age, prevalence of anemia, and cognitive performance of adolescent females.

Variable	Baseline			Postintervention		
	Intervention (*n* = 83)	Control (*n* = 68)	*p* value	Intervention (*n* = 83)	Control (*n* = 68)	*p* value
Age in years (mean ± SD)	12.87 ± 1.89	11.78 ± 1.68	<0.001			
Mean hemoglobin (g/dl) and cognitive test score						
Hb levels (g/dl) (mean ± SD)	11.74 ± 2.09	11.52 ± 2.076	0.521	12.39 ± 1.41	11.45 ± 1.58	<0.001
Cognitive test score (mean ± SD)	24.19 ± 6.63	22.57 ± 5.82	0.117	26.19 ± 6.19	23.40 ± 5.75	0.005
Prevalence of anemia (%)						
Anemic	50.6	50	0.411	38.6	61.8	0.005
Not anemic	49.4	50	0.031	61.4	38.2	
Cognitive performance (%)						
Pass	83.1	79.4	0.002	90.4	79.4	0.058
Fail	16.9	20.6	0.541	9.6	20.6	

Note: the pass mark for the cognitive test score was ≥50%. *p* value is significant at *p* ≤ 0.05.

**Table 2:** Clinical and biochemical characteristics of the participants.

Variable	Baseline		*p* value	Postintervention		*p* value
	Control (*n* = 68)	Intervention (*n* = 83)		Control (*n* = 68)	Intervention (*n* = 83)	
Malaria (%)						
No	41.2	57.8	0.042	88.2	81.9	0.283
Yes	58.8	42.2		11.8	18.1	
Urinary blood loss (%)						
Negative	88.2	71	0.062	97	91.6	0.518
Normal	7.4	22.9		1.5	3.6	
Trace	4.4	3.6		0	1.2	
Moderate	0	2.4		1.5	1.2	
Menstruating	0	0		0	2.4	
Urinary protein loss (%)						
Negative	35.3	19.3	0.141	61.7	34.9	0.003
Normal	10.3	13.3		10.3	4.8	
Trace	47	50.6		25	37.3	
Small	5.9	6		1.5	10.8	
Moderate	1.5	9.6		1.5	6	
Large	0	1.2		0	3.6	
Menstruating	0	0		0	2.4	

Note: urinary blood loss: normal < 10 cacells/*μ*l, trace = 10 cacells/*μ*l, and moderate = 80 cacells/*μ*l; urinary protein loss: normal < 0.3, trace = 0.3 g/l, small = 1 g/l, moderate = 3 g/l, and large ≥ 20. *p* value is significant at *p* ≤ 0.0.

**Table 3:** Consumption of the nine aggregated food groups by the participants.

Food group	Intervention (*n* = 83) Frequency (%)	Control (*n* = 68) Frequency (%)	*p* value
Starchy staples	83 (100)	68 (100)	
Meat/fish	55 (66.3)	52 (76.5)	0.188
Egg	12 (14.5)	6 (8.8)	0.057
Milk and milk products	8 (9.6)	9 (13.2)	0.953
Legumes, nuts, and seeds	58 (69.9)	29 (42.6)	<0.0001
Organ meat	0 (0)	0 (0)	
Dark green leafy vegetable	10 (12.0)	33 (48.5)	<0.0001
Other vitamin A-rich fruits	1 (1.2)	0 (0)	0.204
Other fruits and vegetables	77 (92.8)	56 (82.4)	0.134

*p* value is significant at *p* ≤ 0.05.

**Table 4:** The impact of turkey berry-fortified biscuits on hemoglobin level and cognitive performance.

Variable (*n* = 83)	Coefficient	*p* value
Hemoglobin level (g/dl)	0.945	0.001
Cognitive performance	2.796	0.005

**Table 5:** Mineral content (mg/kg) of placebo and turkey berry-fortified biscuits.

Minerals	*N*	Placebo	Turkey berry-biscuit	*p* value
Ca	3	1170.99 ± 0.17^a^	2627.11 ± 0.34^b^	<0.001
Fe	3	10.88 ± 0.01^a^	17.94 ± 0.01^b^	<0.001
K	3	3025.31 ± 1.22^a^	8126.12 ± 3.88^b^	<0.001
Mg	3	551.73 ± 0.04^a^	1088.23 ± 0.17^b^	<0.001
Zn	3	19.37 ± 0.01^a^	20.88 ± 0.06^b^	<0.001

Placebo biscuits (100% wheat flour) and turkey berry-fortified biscuits (80% wheat flour : 20% turkey berry). Values are expressed as mean ± SD (minerals were analyzed in triplicate).

## Data Availability

The data that support the findings of this study are available from the corresponding author upon reasonable request.
